# Brominated Flame Retardants and Other Persistent Organohalogenated Compounds in Relation to Timing of Puberty in a Longitudinal Study of Girls

**DOI:** 10.1289/ehp.1408778

**Published:** 2015-05-08

**Authors:** Gayle C. Windham, Susan M. Pinney, Robert W. Voss, Andreas Sjödin, Frank M. Biro, Louise C. Greenspan, Susan Stewart, Robert A. Hiatt, Lawrence H. Kushi

**Affiliations:** 1 California Department of Public Health, Richmond, California, USA; 2 University of Cincinnati College of Medicine, Cincinnati, Ohio, USA; 3 Impact Assessment Inc., La Jolla, California, USA; 4 Centers for Disease Control and Prevention, Atlanta, Georgia, USA; 5 Kaiser Permanente, San Francisco, California, USA; 6 Department of Public Health Sciences, University of California, Davis, Davis, California, USA; 7 Department of Epidemiology and Biostatistics, University of California, San Francisco, San Francisco, California, USA; 8 Division of Research, Kaiser Permanente, Oakland, California, USA

## Abstract

**Background:**

Exposure to hormonally active chemicals could plausibly affect pubertal timing, so we are investigating this in the Breast Cancer and the Environment Research Program.

**Objectives:**

Our goal was to examine persistent organic pollutants (POPs) in relation to pubertal onset.

**Methods:**

Ethnically diverse cohorts of 6- to 8-year-old girls (*n* = 645) provided serum for measure of polybrominated diphenyl ethers (PBDEs), polychlorinated biphenyls (PCBs), organochlorine pesticides (OCPs), and lipids. Tanner stages [breast (B) and pubic hair (PH)], and body mass index (BMI) were measured at up to seven annual clinic visits. Using accelerated failure time models, we calculated time ratios (TRs) for age at Tanner stages 2 or higher (2+) and POPs quartiles (Q1–4), adjusting for confounders (race/ethnicity, site, caregiver education, and income). We also calculated prevalence ratios (PRs) of Tanner stages 2+ at time of blood sampling.

**Results:**

Cross-sectionally, the prevalence of B2+ and PH2+ was inversely related to chemical serum concentrations; but after adjustment for confounders, only the associations with B2+, not PH2+, were statistically significant. Longitudinally, the age at pubertal transition was consistently older with greater chemical concentrations; for example: adjusted TR for B2+ and Q4 for ΣPBDE = 1.05; 95% CI: 1.02, 1.08, for ΣPCB = 1.05; 95% CI: 1.01, 1.08, and for ΣOCP = 1.10; 95% CI: 1.06, 1.14, indicating median ages of about 6 and 11 months older than least exposed, and with similar effect estimates for PH2+. Adjusting for BMI attenuated associations for PCBs and OCPs but not for PBDEs.

**Conclusions:**

This first longitudinal study of puberty in girls with serum POPs measurements (to our knowledge) reveals a delay in onset with higher concentrations.

**Citation:**

Windham GC, Pinney SM, Voss RW, SjÖdin A, Biro FM, Greenspan LC, Stewart S, Hiatt RA, Kushi LH. 2015. Brominated flame retardants and other persistent organohalogenated compounds in relation to timing of puberty in a longitudinal study of girls. Environ Health Perspect 123:1046–1052; http://dx.doi.org/10.1289/ehp.1408778

## Introduction

The trend toward earlier age at pubertal development has raised the concern that exogenous exposures may be contributing factors (Herman-Giddens et al. 1997; Euling et al. 2008a, 2008b). This potential association, which had been little studied, and the association of early menarche with increased risk of breast cancer and other adverse outcomes (Bernstein 2002; Golub et al. 2008) led to design of a longitudinal study of determinants of puberty within the Breast Cancer and the Environment Research Program (BCERP). BCERP includes transdisciplinary research collaborations integrated across biologic, epidemiologic, and community outreach projects. Of primary interest are exposures to endocrine disruptors in young girls before breast development or other key pubertal milestones.

During the last few decades, evidence has grown about the health effects of environmental contaminants that have hormonally active properties. Although some of the chemicals of concern [dichlorodiphenyltrichloroethane (DDT) and polychlorinated biphenyls (PCBs)] were banned decades ago in many industrialized countries, with subsequent declines in serum concentrations, they do persist in the environment, and DDT is still used elsewhere (Eskenazi et al. 2009; Sjödin et al. 2004a, 2014). In contrast, increasing levels of the flame retardants polybrominated diphenyl ethers (PBDEs) were found in the environment and human biospecimens in the last 10–15 years (Schecter et al. 2005; Sjödin et al. 2004a). Despite phaseouts since, PBDEs persist in the environment and products containing them, so body burdens may decline slowly (Sjödin et al. 2014). Exposure to these persistent compounds in children occurs primarily through the diet (including breastfeeding), as well as dust or inhalation for PBDEs (Lorber 2008).

Human health effect studies of PBDEs have only relatively recently been reported, and animal studies show thyroid hormone disruption and neurodevelopmental deficits (reviewed by Darnerud et al. 2001). Prenatal PBDE serum concentrations were inversely associated with thyroid hormone levels, infant birth weight, and scores on indices of childhood neurodevelopment in a few study cohorts (Chevrier et al. 2011; Harley et al. 2011; Herbstman et al. 2010). PBDE serum concentrations were also positively associated with time to pregnancy (Harley et al. 2010). The single study of puberty, based on National Health and Nutrition Examination Survey (NHANES) data, found that higher serum PBDE concentrations in adolescent girls (12–19 years of age) were associated with slightly younger retrospectively reported ages at menarche (Chen et al. 2011).

In contrast to PBDEs, more work has been conducted on the health effects of organochlorine pesticides (OCPs). DDT or its metabolite, dichlorodiphenyldichloroethylene (DDE), have been associated with decreased fecundability, spontaneous abortion, preterm birth, small for gestational age, impaired neurodevelopment, and breast cancer (reviewed by Eskenazi et al. 2009). Furthermore, a few studies have reported associations with altered menstrual function, including lower urinary estrogen or progesterone levels, perhaps contributing to some of these adverse reproductive outcomes (Perry et al. 2006; Windham et al. 2005). A handful of studies have examined puberty in girls, but with inconsistent results, based primarily on cross-sectional or retrospective assessment of exposure and/or of age at menarche (Denham et al. 2005; Den Hond et al. 2011; Ouyang et al. 2005; Vasiliu et al. 2004; Wolff et al. 2008).

Similarly, in humans, PCBs have been associated with reduced fecundability, alterations in menstrual cycle function, thyroid hormone disruption, low birth weight or gestational age, deficits in neurodevelopment, and growth alterations [Agency for Toxic Substances and Disease Registry (ATSDR) 2000, 2011]. Not all studies produce consistent results, and comparisons are complicated by the number of congeners examined and their potentially differing mechanisms, as well as timing of exposure. The puberty studies have tended to report inconsistent results among both girls and boys (Denham et al. 2005; Den Hond et al. 2011; Korrick et al. 2011; Vasiliu et al. 2004).

Pubertal development is the result of a series of precisely timed endocrine events. Because the reproductive system does not fully mature until late puberty, this time in development is a window of susceptibility to chemicals that affect the endocrine system, potentially resulting in alterations of development or later reproductive health (Golub et al. 2008). Because there were few prior studies, with generally inconsistent results, our goal was to examine whether peri-pubertal concentrations of persistent organic pollutants (POPs) are associated with age at pubertal onset, as measured by breast and pubic hair development, in the first longitudinal study of girls with this type of data, to our knowledge.

## Materials and Methods

*Study population.* The BCERP includes a cohort study of girls, recruited at ages 6–8 years in 2004–2007, and followed annually to measure onset and progression of pubertal maturation. As reported previously (Biro et al. 2010, 2013), the study was conducted at three sites, using consistent methods, although serum was routinely collected only at two: Kaiser Permanente in the San Francisco Bay Area (“California” site), and Cincinnati Children’s Hospital/ University of Cincinnati, Ohio (“Ohio” site). Eligibility criteria also included no underlying endocrine-associated medical conditions, such as thyroid disease. The sampling frame was defined as age-eligible girls in Kaiser Permanente membership files at enrollment and birth in San Francisco Bay Area clinics, and at selected schools in the greater Cincinnati area (Biro et al. 2010, 2013). Written informed consent was obtained from parent or guardian and child assent was obtained, as approved by the study sites’ institutional review boards. No personally identifiable information was available to the Centers for Disease Control and Prevention (CDC) or California Department of Public Health (CDPH) researchers.

*Sources of data.* In-person clinic visits, conducted annually in California and semi-annually in Ohio, included child anthropometry and pubertal assessment. Collection of information on demographics, reproductive and child health history, and a variety of other factors potentially related to puberty was conducted annually, using comparable questionnaires, answered by a primary caregiver. For this report, most variables used were from study enrollment. Girl’s race/ethnicity was classified into mutually exclusive categories in the following priority order: black (regardless of ethnicity), Hispanic (any race other than black), Asian or Pacific Islander (non-Hispanic), and white or other (non-Hispanic). Other potential covariates obtained from questionnaires included household income, education of the primary caregiver (the majority were the mother), maternal age at delivery and menarche, and breastfeeding duration.

Pubertal maturation was determined through standardized methods based on Tanner staging (Biro et al. 2010). At each visit, breast (B1–B5) and pubic hair (PH1–PH5) stages were assessed by inspection (and palpation for breast stage). Examiners were trained and overseen by an experienced pediatric endocrinologist at each site, following a written protocol developed jointly, with photographs that demonstrated the maturation stages. Based on comparison to a master trainer that visited each site, inter-rater reliability was considered substantial, with 87% agreement or a kappa of 0.67 (Biro et al. 2010). Height and weight were measured using calibrated scales and stadiometers by trained examiners. Body mass index (BMI) was calculated as weight (kilograms)/height (meters) squared and classified into percentiles using age- and sex-specific CDC growth charts (CDC 2000), with ≥ 85th percentile indicating overweight (and obese ≥ 95th). Clinical examination data were included in this analysis for up to 7 years (median, 5 years of follow-up). This study had a high retention, with 87% still seen in year 3 and about 72% in year 7.

Blood samples were collected using materials and procedures provided by the CDC laboratory (Windham et al. 2010) during the first few study years, with the earliest sample for each girl (who provided one or more) submitted for measurement. The Ohio site generated samples from 295 girls and the California site from 350 for determining the concentrations of 10 PBDE congeners (plus 2,2´,4,4´,5,5´-hexabromobiphenyl), 35 PCB congeners, and 9 organochlorine pesticides [*o*,*p*´- and *p*,*p*´-DDT, the metabolite *p*,*p*´-DDE, hexachlorobenzene (HCB), β- and γ-hexachlorocyclohexane (HCH), oxychlordane, *trans*-nonachlor, and mirex] by highly sensitive gas chromatography–isotope dilution high-resolution mass spectrometry (Sjödin et al. 2004b). Method detection limits (MDL) were determined individually for each serum sample and analyte, dependent on volume and blank samples, and were typically in the sub-to-low nanograms per gram lipid range (medians, 0.5–7.8), except DDE (median, 39.6 ng/g lipid) (see Supplemental Material, Table S1). Concentrations in this report are lipid-adjusted (nanograms per gram lipid weight), with lipids determined using commercially available kits (Roche Diagnostic Corp) for total triglycerides and total cholesterol.

*Data analysis.* Serum samples were provided by 645 girls, but we excluded 2 with missing lipid values and another 14 missing key covariate data, leaving 623–629 girls for primary models, depending on the number with data for each chemical. Here we focus on analytes for which > 60% of samples were above the MDL (Windham et al. 2010); for measurements below the MDL, we substituted the value MDL divided by the square root of 2. We also conducted a sensitivity analysis excluding values from samples of low weight because this can result in high MDLs (e.g., 55 samples < 1 g or 18 samples < 0.5 g). We summed the concentrations of the six most frequently detected PBDE congeners (BDEs 28, 47, 99, 100, 153, 154; labeled ΣPBDE) and the six most frequently detected PCB congeners [CBs 99, 118, 138/158 (co-eluted), 153, 170, and 180; labeled ΣPCB]. We also summed the three OCPs—HCB, *trans*-nonachlor, and DDE—for ease of analyses, with DDE representing the bulk. These sums and individual congeners (or pesticides) were categorized into quartiles or examined as natural log-transformed continuous variables and their interquartile ranges (IQRs). Trends were evaluated across quartile effect estimates, treating the quartiles as ordinal variables (1–4), and evaluating the continuous measure (IQR), where examined. Throughout, *p* < 0.05 is considered statistically significant. To examine potential additive effects, a simple index score was created for each girl by summing the quartile values (e.g., 1–4) of each of her three chemical group sums and then categorizing into low (score 3–6), medium (score 7–9), and high (10–12) POP levels.

Initially, we conducted a cross-sectional analysis, examining the proportion of girls who had reached pubertal onset, defined as Tanner stage 2 or greater (or B2+, PH2+) at the time of blood draw, when most girls were 7 or 8 years old. Demographic variables were examined by geometric mean (GM) concentrations and the percent pubertal to evaluate potential confounders. We calculated adjusted prevalence ratios (aPRs) and 95% confidence intervals (CIs) by Poisson regression for Tanner stage 2+ versus stage 1 and quartiles of the serum POPs, adjusting for variables that were identified of *a priori* interest, from the comparison of crude GMs, or that changed the effect estimates by > 10% (race/ethnicity, site, household income, caregiver education, as well as age and BMI at blood draw; see categorizations in Table 1). History of breastfeeding was not included because it provides a source of exposure, but was examined in a sensitivity analysis (none or < 1 month, 1–4 months, > 4 months).

**Table 1:** Distribution, crude time ratios (TRs), and 95% CIs for pubertal development (stage 2+ vs. 1) among girls who provided serum samples, by characteristics at enrollment.

Variable	*n*^*a*^ (%)	Breast [TR (95% CI)]	Pubic hair [TR (95% CI)]
Site
California	350 (54.3)	1.06 (1.04, 1.09)**	1.01 (0.98, 1.03)
Ohio	295 (45.7)	(reference)	(reference)
Child race/ethnicity
Asian	44 (6.8)	1.02 (0.98, 1.07)	1.07 (1.02, 1.12)**
Black	170 (26.4)	0.91 (0.88, 0.93)**	0.88 (0.86, 0.90)**
Hispanic	97 (15.0)	1.00 (0.97, 1.04)	0.97 (0.94, 1.00)
White	334 (51.8)	(reference)	(reference)
Age (years)
> 8	19 (3.00)	0.95 (0.89, 1.01)	0.99 (0.93, 1.06)
7–8	430 (66.7)	1.00 (0.97, 1.02)	0.98 (0.95, 1.01)
< 7	196 (30.4)	(reference)	(reference)
BMI (CDC percentiles)
≥ 85th	188 (29.2)	0.93 (0.90, 0.95)**	0.94 (0.91, 0.96)**
< 85th	457 (70.9)	(reference)	(reference)
Yearly income
< $50,000	183 (28.7)	0.91 (0.89, 0.94)**	0.92 (0.89, 0.94)**
$50,000–$100,000	222 (34.8)	0.96 (0.93, 0.98)**	0.95 (0.93, 0.98)**
≥ $100,000	233 (36.5)	(reference)	(reference)
Caregiver education
High school	92 (14.4)	1.00 (0.97, 1.03)	1.00 (0.96, 1.03)
Some college	201 (31.4)	0.96 (0.94, 0.99)**	0.96 (0.94, 0.99)**
Bachelor’s or greater	348 (54.3)	(reference)	(reference)
Birth mother age at menarche (years)
< 12	198 (35.3)	0.94 (0.91, 0.97)**	0.94 (0.91, 0.97)**
12–13.9	231 (41.2)	0.97 (0.94, 1.00)*	0.98 (0.95, 1.01)
≥ 14	132 (23.5)	(reference)	(reference)
Birth mother age at delivery (years)
< 25	112 (17.4)	0.94 (0.91, 0.98)**	0.95 (0.91, 0.98)**
25 to 29	129 (20.0)	0.96 (0.93, 0.99)*	0.99 (0.95, 1.02)
30 to 34	205 (31.8)	0.99 (0.97, 1.02)	1.02 (0.99, 1.05)
≥ 35	198 (30.8)	(reference)	(reference)
Abbreviations: BMI, body mass index; CI, confidence interval; TR, time ratio. Some CIs = 1.00 due to rounding, even if *p*-value is significant.^***a***^Some totals are < 645 due to missing values; in particular, mother’s age at menarche was missing for 84. **p < 0.01, *p < 0.05, compared with referent group.			

After additional years of follow-up during which most girls had reached pubertal onset, we conducted longitudinal analyses. The association of each exposure with age at onset of B2+ or PH2+ was evaluated in separate accelerated failure time models using a Weibull distribution, with left and right censoring to account for pubertal transitions taking place before or after the period of observation, and interval censoring to account for pubertal transitions between exam visits (SAS Proc Lifereg, SAS version 9.3; SAS Institute Inc.). For girls who reached Tanner stage 2+ during observed follow-up visits, the interval was defined as the period from the last exam visit consistently at stage 1 to the first visit where the girl was observed to be consistently at stage 2 or greater (e.g., no regression to stage 1 in a subsequent visit). The time ratios (TRs) calculated compare the median age at onset among girls with the characteristic of interest, or the exposure, to girls in the reference category. With typical median ages of B2+ and PH2+ between 9 and 10 years old, small TRs can reflect a relatively large difference in age (e.g., 10.5 years/10 years = 1.05, representing 5% later onset or a 6-month lag). To calculate differences in median age from multivariate models, we used exp{inter-cept + scale × ln[ln(2)]}, and multiplied that by the TR for the exposure group of interest for comparison to girls represented by the referent group for all covariates. This yielded results very similar to those from simply multiplying the TR by the crude group medians (118 months for B2+ and 125 for PH2+) because distributions are tight. The covariates included in the cross-sectional analysis were also used to calculate adjusted TRs (aTR), except that age at blood draw was determined not to be a confounder by the change-in-estimate method (and girls’ ages are inherent in these models). We examined BMI at several different time points, but because they were highly correlated with each other (within individuals, Pearson’s *r* > 0.87 for all pairwise comparisons across eight time points), yielding very similar model results, we included BMI at the blood draw visit to best account for a possible pharmacokinetic (or “dilution”) effect. Because BMI may be on the causal pathway, mediation analyses were conducted, running models with and without BMI. To assess differential effects of chemical exposure by BMI status (overweight or “high,” vs. “healthy,” or < 85th percentile), we tested interaction terms in models of age at onset of B2 or PH2 that contained main effects for BMI (one term), for exposure (three terms), and for their interaction (three terms), as well as the covariates. Adjusted TRs with 95% CI were constructed for quartiles of exposure by BMI level (maintaining Q1 as the reference within each BMI group), and for high versus healthy BMI at the lowest quartile of exposure; a chi-square statistic with 3 degrees of freedom was used to assess the statistical significance of the interaction.

## Results

This sample of girls was racially diverse and nearly one-third had a BMI at enrollment in the overweight or obese range (Table 1). At the time of the blood draw, girls ranged in age from 6 to 9 years, but most were 7 and 8. About 80% of girls provided blood specimens (the only girls included in this analysis) and they were demographically representative of all girls at these two sites (data not shown). At enrollment, about 11% of the girls had reached B2+ and 9% PH2+, and by the end of the follow-up period, 89% had reached B2+ and 82% PH2+. As recently reported (Biro et al. 2013), modeled median age at onset of B2+ in the overall three-site cohort was 8.8, 9.3, 9.7, and 9.7 years for black, Hispanic, white, and Asian participants, respectively, consistent with the TR of 0.91 (95% CI: 0.88, 0.93) also indicating an earlier age of B2+ onset among non-Hispanic black, compared with white, girls in this subsample (Table 1). Onset of pubic hair development also varied by race/ethnicity, with earlier age for blacks, but older for Asians compared with whites. Pubertal onset was earlier among girls in Ohio (for breast development), those with higher BMI, living in households with lower income, and whose mothers had an earlier age at delivery or menarche (Table 1).

Information about the distributions (percent > MDL, GMs, quartile cut points) of the chemicals and sums are presented in Supplemental Material, Table S1, and were described in detail previously (Windham et al. 2010). The adjusted GM levels of the POPs were generally higher in the girls from California compared with Ohio and NHANES 2003–2004, and in girls of “healthy” BMI, as well as varying by other demographic variables (such as race or maternal age) for each chemical class (Windham et al. 2010).

The proportion having reached B2+ at time of blood draw decreased from roughly 30–40% in the first quartile for all chemical groups to 5–12% in the fourth quartiles, yielding significant PRs in crude and adjusted models (without BMI) (Table 2) and significant trends for ΣPCBs and ΣPBDEs, but not for OCPs. Adding BMI to the model attenuated associations; only the ΣPCB and B2+ showed a monotonic decrease (trend test *p*-value < 0.05) and significant PRs for the 3rd and the 4th (aPR = 0.38; 95% CI: 0.18, 0.80) quartiles (Table 2). As for B2+, inverse associations were also seen for PH2+ with PCBs and OCPs crudely, but no significant trends or PRs after adjustment (Table 2).

**Table 2:** Crude and adjusted prevalence ratios (PRs) and 95% CIs for Tanner stage 2+ vs. 1 at time of blood draw.

Tanner measure	Lipid-adjustedchemical class sum	Quartile	Percent Tanner 2+	Crude PR (95% CI)	Adjusted^*a*^ PR (95% CI)	Adjusted^*b*^ PR (95% CI)
Breast development	ΣPCB	Q1	38.6	(reference)**	(reference)**	(reference)*
		Q2	22.0	0.58 (0.41, 0.83)	0.64 (0.45, 0.91)	0.83 (0.60, 1.15)
		Q3	14.5	0.38 (0.25, 0.59)	0.45 (0.29, 0.68)	0.66 (0.44, 0.99)
		Q4	5.0	0.13 (0.07, 0.27)	0.21 (0.10, 0.45)	0.38 (0.18, 0.80)
	ΣPBDE	Q1	27.3	(reference)*	(reference)*	(reference)^#^
		Q2	22.6	0.79 (0.54, 1.17)	0.85 (0.60, 1.21)	1.02 (0.72, 1.44)
		Q3	17.5	0.63 (0.41, 0.96)	0.70 (0.48, 1.04)	0.79 (0.54, 1.15)
		Q4	12.6	0.45 (0.27, 0.73)	0.51 (0.32, 0.83)	0.68 (0.44, 1.05)
	ΣOCP	Q1	42.4	(reference)	(reference)	(reference)
		Q2	16.5	0.35 (0.23, 0.53)	0.48 (0.31, 0.75)	0.69 (0.44, 1.07)
		Q3	11.3	0.26 (0.16, 0.42)	0.41 (0.24, 0.70)	0.71 (0.43, 1.17)
		Q4	9.9	0.22 (0.13, 0.37)	0.44 (0.23, 0.84)	0.89 (0.46, 1.73)
Pubic hair development	ΣPCB	Q1	25.2	(reference)	(reference)	(reference)
		Q2	13.5	0.56 (0.35, 0.92)	0.72 (0.44, 1.16)	0.76 (0.46, 1.25)
		Q3	10.2	0.42 (0.24, 0.72)	0.60 (0.35, 1.03)	0.64 (0.36, 1.14)
		Q4	8.3	0.34 (0.19, 0.61)	0.59 (0.32, 1.10)	0.65 (0.33, 1.27)
	ΣPBDE	Q1	15.8	(reference)	(reference)	(reference)
		Q2	14.6	0.98 (0.58, 1.67)	0.87 (0.53, 1.42)	0.91 (0.55, 1.51)
		Q3	13.3	0.89 (0.51, 1.54)	0.73 (0.42, 1.27)	0.76 (0.44, 1.32)
		Q4	12.9	0.83 (0.47, 1.45)	0.65 (0.37, 1.15)	0.70 (0.39, 1.26)
	ΣOCP	Q1	20.6	(reference)	(reference)	(reference)
		Q2	13.0	0.61 (0.36, 1.03)	0.74 (0.44, 1.26)	0.81 (0.47, 1.39)
		Q3	10.9	0.52 (0.30, 0.90)	0.65 (0.35, 1.22)	0.76 (0.40, 1.46)
		Q4	12.0	0.56 (0.33, 0.95)	0.76 (0.38, 1.53)	0.92 (0.43, 1.97)
Abbreviations: ΣOCP, sum of organochlorinated pesticides; ΣPBDE, sum of polybrominated diphenyl ethers; ΣPCB, sum of polychlorinated biphenyls; Q1–4, quartiles 1–4.^***a***^Models include race, household income, caregiver education, site, and age at blood draw. ^***b***^Adds BMI at blood draw to covariates in the first model. **p < 0.01, *p < 0.05, ^#^*p* < 0.1 represent *p*-values for trend test across quartiles (ordinal variable) within chemical class.						

Longitudinally, pubertal transition (B2+ or PH2+) was later across increasing quartiles or the continuous measures of all three chemical groups (Table 3). The TRs were only slightly affected by adjustment, and significant trends persisted, as indicated by the IQR TRs. Adding BMI to the models attenuated the TRs and trends, although some associations were still significant. For example, at the ΣPBDE fourth quartile, the aTRs were 1.04 for B2+ (95% CI: 1.01, 1.07) and 1.05 for PH2+ (95% CI: 1.01, 1.08) (Table 3), indicating median ages of 4.7 and 6.4 months older than the least exposed, respectively. Age at breast development was also older among girls with higher pesticide concentrations (Q4 aTR = 1.04; 95% CI: 1.00, 1.09), but with a plateau versus a dose pattern (Table 3). After full adjustment (with BMI), the IQR TRs were only significantly associated with ΣPBDE (aTR for B2+ = 1.08; 95% CI: 1.00, 1.14 and for PH2+ = 1.11; 95% CI: 1.04, 1.19).

**Table 3:** Crude and adjusted time ratios (TRs) and 95% CIs of transition to Tanner stage 2+ by quartiles and interquartile range (IQR) of each chemical class sum.

Lipid-adjusted chemical class	Crude TR		Adjusted^*a*^ TR		Adjusted^*b*^ TR	
	Breast	Pubic hair	Breast	Pubic hair	Breast	Pubic hair
ΣPCB
Q2	1.04 (1.01, 1.07)**	1.05 (1.02, 1.09)**	1.04 (1.01, 1.07)*	1.04 (1.01, 1.07)*	1.01 (0.98, 1.04)	1.02 (0.98, 1.05)
Q3	1.06 (1.03, 1.10)**	1.07 (1.04, 1.11)**	1.04 (1.01, 1.08)*	1.04 (1.01, 1.08)**	1.01 (0.98, 1.04)	1.01 (0.98, 1.05)
Q4	1.07 (1.04, 1.11)**	1.07 (1.04, 1.11)**	1.05 (1.01, 1.08)**	1.05 (1.01, 1.08)**	0.99 (0.96, 1.03)	1.01 (0.97, 1.04)
IQR^*c*^ (44.6 ng/g)	1.11 (1.04, 1.16)**	1.11 (1.04, 1.16)*	1.06 (1.00, 1.12)*	1.07 (1.04, 1.12)**	0.98 (0.93, 1.04)	1.01 (0.96, 1.08)
ΣPBDE
Q2	1.03 (1.00, 1.07)*	1.02 (0.99, 1.05)	1.03 (1.00, 1.06)	1.01 (0.98, 1.04)	1.02 (0.99, 1.05)	1.01 (0.98, 1.04)
Q3	1.03 (0.99, 1.06)	1.04 (1.00, 1.07)*	1.03 (1.00, 1.06)	1.03 (1.00, 1.07)*	1.02 (0.99, 1.05)	1.03 (1.00, 1.06)
Q4	1.05 (1.02, 1.08)**	1.05 (1.01, 1.08)**	1.05 (1.02, 1.08)**	1.05 (1.02, 1.09)**	1.04 (1.01, 1.07)*	1.05 (1.01, 1.08)**
IQR^*c*^ (75.6 ng/g)	1.09 (1.00, 1.19)*	1.10 (1.04, 1.19)*	1.10 (1.04, 1.19)**	1.13 (1.04, 1.19)**	1.08 (1.00, 1.14)*	1.11 (1.04, 1.19)**
ΣOCP
Q2	1.09 (1.05, 1.12)**	1.06 (1.03, 1.10)**	1.07 (1.04, 1.11)**	1.06 (1.02, 1.09)**	1.05 (1.02, 1.08)**	1.04 (1.01, 1.08)*
Q3	1.12 (1.09, 1.16)**	1.08 (1.04, 1.11)**	1.09 (1.05, 1.12)**	1.07 (1.03, 1.11)**	1.04 (1.00, 1.07)*	1.04 (1.00, 1.08)*
Q4	1.14 (1.11, 1.17)**	1.07 (1.04, 1.11)**	1.10 (1.06, 1.14)**	1.08 (1.04, 1.13)**	1.04 (1.00, 1.09)*	1.04 (0.99, 1.09)
IQR^*c*^ (153.0 ng/g)	1.30 (1.22, 1.41)**	1.14 (1.05, 1.22)**	1.18 (1.10, 1.28)**	1.13 (1.05, 1.22)**	1.06 (0.95, 1.16)	1.03 (0.95, 1.10)
Abbreviations: IQR, interquartile range of continuous variable; ΣOCP, sum of organochlorinated pesticides; ΣPBDE, sum of polybrominated diphenyl ethers; ΣPCB, sum of polychlorinated biphenyls; Q1–4, quartiles 1–4. ^***a***^Models include race/ethnicity, household income, caregiver education, site, with Q1 as the reference group for models with Qs 2–4. ^***b***^Adds BMI at serum draw to covariates in the first model. ^***c***^Modeled with chemical as continuous variable and TR calculated for IQR. **p < 0.01. *p < 0.05.						

Including all three chemical group sums as continuous variables in one model, the TR for the IQR of the ΣPCB and B2+ was attenuated (aTR = 1.01; 95% CI: 0.96, 1.08) but the other two chemical classes had TRs similar to those in the separate models. Adding covariates (including BMI) to this model, the IQR TR for ΣPCBs and B2+ was further attenuated, becoming inverse (0.93; 95% CI: 0.86, 1.00, *p* = 0.04), but strengthened for B2+ and OCPs (1.15; 95% CI: 1.00, 1.28, *p* = 0.02), while remaining similar for ΣPBDEs (aTR = 1.07; 95% CI: 1.00, 1.14, *p* = 0.04). The IQR TRs for PH2+ were similar to those from separate chemical models (data not shown).

In sensitivity analyses, adding breastfeeding duration to the quartile models did not affect patterns observed or statistical significance, and (separately) excluding girls with low specimen weights produced nearly identical results (data not shown).

By specific congeners, the associations with pubertal onset (B2+ or PH2+) were fairly similar across the BDE congeners in crude (data not shown) and adjusted models with some patterns of monotonic increase (Table 4; see also Supplemental Material, Table S2). BDE-154 was the only congener not associated with either end point (Q4 vs. Q1) in adjusted (without BMI) models, whereas BDE-153 was most strongly associated with both end points (aTRs = 1.08 for Q4 vs. Q1) (Table 4). Adding BMI did not affect these BDE congener results greatly, although slightly attenuated results for BDE-153 and BDE-154 became significantly associated with PH2+ at Q4. Across the PCB congeners, adjusted TRs varied only slightly between the six in the PCB sum with less pattern of a monotonic increase (see Supplemental Material, Table S2), but were strongest and consistently associated with both end points for CBs 138/158 and 180, at Q4 versus Q1 (Table 4). Adding BMI to the PCB congener models attenuated all TRs to near null, nonsignificant values (Table 4; see also Supplemental Material, Table S2). Among the pesticides, DDE and HCB had similar Q4 versus Q1 associations with older age at pubertal onset (both end points) and patterns of increase across the quartiles, but *trans*-nonachlor less so (Table 4; see also Supplemental Material, Table S2). Adjusting for BMI also attenuated these TRs, disrupting dose patterns, and leaving significant associations only for breast development (with DDE or HCB).

**Table 4:** Adjusted*^a^* time ratios (TRs) and 95% CIs of transition to Tanner stage 2+ for individual congeners: Q4 versus Q1.*^b^*

Lipid-adjusted congener/OCP	Adjusted^*a*^		Adjusted^*a*^ including BMI	
	Breast	Pubic hair	Breast	Pubic hair
PCBs
PCB-99	1.04 (1.00, 1.07)*	1.03 (1.00, 1.07)*	0.98 (0.95, 1.02)	1.00 (0.97, 1.04)
PCB-118	1.04 (1.00, 1.07)*	1.04 (1.01, 1.08)*	0.99 (0.96, 1.03)	1.01 (0.98, 1.05)
PCB-153	1.03 (1.00, 1.07)*	1.05 (1.01, 1.08)**	0.99 (0.95, 1.02)	1.01 (0.97, 1.05)
PCB-170	1.02 (0.99, 1.06)	1.04 (1.01, 1.08)*	0.98 (0.94, 1.01)	1.00 (0.97, 1.04)
PCB-180	1.05 (1.01, 1.08)**	1.06 (1.02, 1.09)**	0.99 (0.96, 1.03)	1.02 (0.98, 1.06)
PCB-138/158	1.05 (1.02, 1.09)**	1.05 (1.01, 1.08)**	1.01 (0.98, 1.04)	1.01 (0.98, 1.05)
PBDEs
PBDE-28	1.02 (0.99, 1.06)	1.04 (1.01, 1.07)*	1.02 (0.99, 1.05)	1.04 (1.01, 1.07)**
PBDE-47	1.03 (1.00, 1.07)*	1.04 (1.00, 1.07)*	1.04 (1.01, 1.07)*	1.04 (1.01, 1.07)*
PBDE-99	1.03 (1.00, 1.06)*	1.04 (1.01, 1.07)*	1.04 (1.01, 1.06)*	1.04 (1.01, 1.07)*
PBDE-100	1.05 (1.02, 1.09)**	1.04 (1.01, 1.08)*	1.06 (1.03, 1.09)**	1.04 (1.01, 1.08)**
PBDE-153	1.08 (1.05, 1.12)**	1.08 (1.04, 1.11)**	1.04 (1.01, 1.08)**	1.05 (1.02, 1.09)**
PBDE-154	1.00 (0.97, 1.03)	1.03 (0.99, 1.06)	1.01 (0.98, 1.04)	1.03 (1.00, 1.06)*
OCPs
DDE	1.10 (1.05, 1.14)**	1.08 (1.03, 1.12)**	1.04 (1.00, 1.08)	1.03 (0.99, 1.08)
*trans*-Nonachlor	1.01 (0.98, 1.04)	1.04 (1.01, 1.08)*	0.98 (0.95, 1.01)	1.02 (0.99, 1.05)
Hexachlorobenzene	1.10 (1.06, 1.13)**	1.06 (1.03, 1.09)**	1.04 (1.01, 1.08)*	1.02 (0.98, 1.06)
Abbreviations: BMI, body mass index; DDE, dichlorodiphenyldichloroethene; OCP, organochlorinated pesticides; PBDE, polybrominated diphenyl ethers; PCB, polychlorinated biphenyls. ^***a***^Models include race/ethnicity, household income, caregiver education, site. ^***b***^See Supplemental Material, Table S2, for other quartile TRs. **p < 0.01. *p < 0.05.				

Examining the index score summing across POP groups, similar patterns of significantly later pubertal transition (both end points) with higher scores were seen. The effect estimates did not indicate even later transition with high levels of all three as compared to the individual chemical group Q4 versus Q1 TRs; for example, for B2+ and high score, aTR = 1.06 (95% CI: 1.03, 1.11) and for medium score, aTR = 1.04 (95% CI: 1.01, 1.06). Results were similar for PH2+; high score aTR = 1.05 (95% CI: 1.02, 1.09), and for medium score aTR = 1.03 (95% CI: 1.00, 1.05). Adding BMI to the models attenuated these TRs to 1.02 or less, with no significant associations (data not shown).

Examining interactions with BMI (Table 5), we found interactions for PCBs on both breast (*p* = 0.05) and pubic hair (*p* = 0.02) development that differed from overall associations; among girls with higher BMI, pubertal transition occurred earlier, not later, for those at the highest quartile compared with the lowest (aTR for B2+ = 0.94; 95% CI: 0.88, 1.00 and for PH2+ aTR = 0.93; 95% CI: 0.87, 1.00). Among girls with a healthy BMI, the pattern was for later transition with greater PCB exposure, but only significant for PH2+ (aTR for Q4 = 1.05; 95% CI: 1.01, 1.10) (Table 5). In contrast, the strong interaction between OCPs and BMI on breast development (*p*-value = 0.01) indicated a longer delay associated with exposure in girls with high BMI (Q4 vs. Q1 aTR = 1.15; 95% CI: 1.08, 1.23) than in girls with healthy BMI (aTR = 1.03; 95% CI: 0.99, 1.08). There was no interaction between OCPs and BMI for PH2+ (interaction *p*-value 0.88). Nor was an interaction with BMI seen for PBDEs and either end point. Overall, the effect of earlier transition with higher BMI is reflected in the TRs < 1.0 for high versus healthy BMI among the lowest exposed for each chemical class, generally stronger for B2+ than PH2+ (Table 5).

**Table 5:** Adjusted*^a^* time ratios and 95% CIs for transition to Tanner stage 2+, chemical class, and BMI*^b^* interaction models.

Chemical × BMI group	ΣPCBs		ΣPBDEs		ΣOCPs	
	Breast (*n* = 625)	Pubic hair (*n* = 622)	Breast (*n* = 626)	Pubic hair (*n* = 623)	Breast (*n* = 626)	Pubic hair (*n* = 624)
Interaction *p*‑values
High BMI
Q1	Reference	Reference	Reference	Reference	Reference	Reference
Q2	1.00 (0.95, 1.05)	1.02 (0.97, 1.07)	1.03 (0.98, 1.09)	1.03 (0.98, 1.08)	1.11 (1.06, 1.17)**	1.06 (1.01, 1.11)*
Q3	1.03 (0.97, 1.08)	0.99 (0.94, 1.04)	1.07 (1.02, 1.13)**	1.05 (1.00, 1.11)	1.10 (1.04, 1.17)**	1.07 (1.01, 1.14)*
Q4	0.94 (0.88, 1.00)*	0.93 (0.87, 1.00)	1.06 (1.01, 1.12)*	1.09 (1.04, 1.15)*	1.15 (1.08, 1.23)**	1.05 (0.98, 1.12)
Healthy BMI
Q1	Reference	Reference	Reference	Reference	Reference	Reference
Q2	1.03 (0.99, 1.08)	1.04 (1.00, 1.09)	1.01 (0.98, 1.05)	1.00 (0.97, 1.04)	1.03 (0.99, 1.07)	1.04 (1.00, 1.09)*
Q3	1.02 (0.98, 1.06)	1.05 (1.01, 1.10)*	1.00 (0.97, 1.04)	1.02 (0.98, 1.06)	1.03 (0.99, 1.07)	1.05 (1.00, 1.10)*
Q4	1.03 (0.99, 1.07)	1.05 (1.01, 1.10)*	1.04 (1.00, 1.07)*	1.03 (1.00, 1.07)	1.03 (0.99, 1.08)	1.06 (1.01, 1.11)*
BMI at Q1
High	0.94 (0.90, 0.98)**	1.00 (0.95, 1.04)	0.89 (0.85, 0.93)**	0.93 (0.89, 0.97)**	0.88 (0.84, 0.91)**	0.96 (0.92, 1.00)*
Healthy	Reference	Reference	Reference	Reference	Reference	Reference
Abbreviations: BMI, body mass index; ΣOCP, sum of organochlorinated pesticides; ΣPBDE, sum of polybrominated diphenyl ethers; ΣPCB, sum of polychlorinated biphenyls; Q1–4, quartiles 1–4. To compare the quartile TRs to a single reference group of Q1 in healthy BMI group, multiply the high BMI quartile TRs by the TR for “BMI at Q1 High.” ^***a***^Models include race/ethnicity, household income, caregiver education, site. ^***b***^High BMI is ≥ 85th percentile, Healthy is < 85th percentile. **p < 0.01, *p < 0.05, compared with referent group.						

## Discussion

The results of our study generally indicate that timing of pubertal onset (as measured by both breast and pubic hair development) was delayed in association with higher POPs body burdens, though attenuated when adjusted for BMI. Cross-sectional results were in the same direction for breast, but not pubic hair, development, and were also attenuated by BMI. The delays were most consistent across end points and different models for the PBDEs, not affected by adjusting for the other chemical groups, and strongest for congener BDE-153. Delays in breast development were also seen with hexachlorobenzene and DDE, including after adjustment for BMI. Associations between PCBs and pubertal onset were nearly null after adjustment for BMI, suggesting that BMI may be a causal intermediate. Furthermore, interaction analyses showed that higher PCB concentrations were associated with earlier breast onset among overweight, but not healthy BMI girls. Girls with lower BMI, as examined at a number of time points, tended to have higher body burdens of these POPs (Windham et al. 2010), which may reflect an effect of exposure on reducing growth as found in some, but not all, studies (ATSDR 2000), or pharmacokinetic effects. The observation that adjusting for BMI did not affect associations between PBDEs (summed and individual congeners) and the onset of puberty, and the finding that interactions with BMI varied between the chemical groups, suggest this is not strictly a pharmacokinetic effect.

The one other study that examined female puberty and PBDEs found some evidence for an inverse association with age at menarche, significant only for BDE-47 measured cross-sectionally (Chen et al. 2011). Defining early age at menarche as < 12 years significantly increased risks at the fourth quartile of the PBDE sum as well as several congeners (but not BDE-153), even after adjusting for BMI *z*-score. This study differed from ours in that PBDEs were not measured prepubertally and concentrations were 40–50% lower, as well as examining a later stage of puberty, retrospectively reported among adolescents 12–19 years of age. In contrast, animal studies have suggested a delay in the onset of puberty in females with either peripubertal exposure (Stoker et al. 2004) or gestational exposure (Lilienthal et al. 2006). Most PBDE congeners act as anti-androgens, perhaps affecting pubarche, but also have varying estrogenic effects (Darnerud et al. 2001).

Previous studies of PCBs and OCPs in relation to puberty in girls have yielded inconsistent results, but have used a variety of methods and represent widely varying time frames and body burdens. Among cross-sectional studies, one in a contemporary group of adolescents in Flanders found that higher PCB levels were significantly associated with a delay in menarche, but no association with OCPs, or for either chemical group with Tanner stage (Den Hond et al. 2011). In a small study of Akwesasne Mohawk adolescents whose POP body burdens appear low compared with those of other studies, girls with relatively higher levels of a group of four potentially estrogenic PCB congeners (CBs 52, 70, 101, 187) were more likely to have already reached menarche, but there was no association with other PCB groupings, DDE, or HCB in the same models (Denham et al. 2005). A study in New York (Wolff et al. 2008) found plasma DDE or PCB concentrations were not related to breast or pubic hair stage in 9-year-old girls studied in 1996–1997. However, the girls with lower BMI (< median) and higher PCB concentrations (> median) were less likely to be B2+ than girls with higher BMI and lower PCB concentrations, which is consistent with our results.

Two studies examined maternal (*in utero*) levels in relation to pubertal timing in girls born in the 1950s–1980s, with up to an order of magnitude higher PCB and DDE concentrations than in our study. Examining retrospectively reported (at adolescence or adulthood) age at menarche, earlier age was seen with higher PCB or DDE levels in one study, although not significantly so nor adjusted for other factors (Gladen et al. 2000). The other study found earlier menarche associated with maternal DDE levels, but not PCBs, which was attenuated when controlling for estimated body size at menarche (Vasiliu et al. 2004).

Age at pubertal onset in boys may also be of interest for comparison, particularly a prospective study with several similarities to ours, conducted in Russia. Suggestive evidence was found for delays in puberty with higher dioxin or co-planar PCB levels measured in the boys at enrollment (Korrick et al. 2011), as well as with higher HCB, but not DDE, concentrations and one indicator of pubertal development (testicular volume) (Lam et al. 2014), providing potential support for our findings.

Compared with the previous investigations in girls, our study has numerous strengths. These include *a*) being the first to assess early stages of puberty by clinical examination using standardized measures, *b*) in a large, diverse sample of *c*) prepubertal (mostly) girls followed prospectively. Further, we collected data on a number of covariates, and POP serum levels were measured using state-of-the-art assays. There are also limitations, including that the results may not be generalizable nationwide: The sample draws from urban populations in two geographic locations only, participants may not be representative because they were willing to participate in an ongoing study that included multiple clinic appointments. Residual confounding may also be an issue. We used only a single serum measurement, which may not represent the most susceptible exposure period. However all these compounds are persistent, reflecting long-term exposures, even *in utero* and lactationally, so relative ranking by quartile may remain consistent. Birth cohort studies with long-term follow-up could address this issue.

Our study shows that puberty is delayed among girls with higher serum levels of peri-pubertal POPs, by a magnitude similar to effect estimates of more well-known predictors of pubertal timing, such as BMI and race/ethnicity (Table 1; see also Biro et al. 2013). Although not in the direction to explain general trends of earlier onset of puberty, these results may indicate an effect of exposure to these POPs on the reproductive system during a susceptible period of development that could in turn influence later health end points.

## Supplemental Material

(167 KB) PDF
