# Supplementary material for: Brominated Flame Retardants and Other Persistent Organohalogenated Compounds in Relation to Timing of Puberty in a Longitudinal Study of Girls
Source: Environ Health Perspect. 2015 May 8;123(10):1046–52. doi: 10.1289/ehp.1408778 (PMC4590751; doi:10.1289/ehp.1408778)
Supplement: (167 KB) PDF [file ehp.1408778.s001.acco.pdf]

Environ Health Perspect
doi: 10.1289/ehp.1408778

Note to Readers: *EHP* strives to ensure that all journal content is accessible to all readers. However, some figures and Supplemental Material published in *EHP* articles may not conform to 508 standards due to the complexity of the information being presented. If you need assistance accessing journal content, please contact ehp508@niehs.nih.gov. Our staff will work with you to assess and meet your accessibility needs within 3 working days.

Supplemental Material

Brominated Flame Retardants and Other Persistent Organohalogenated Compounds in Relation to Timing of Puberty in a Longitudinal Study of Girls

Gayle C. Windham, Susan M. Pinney, Robert W. Voss, Andreas Sjodin, Frank M. Biro, Louise C. Greenspan, Susan Stewart, Robert A. Hiatt, and Lawrence H. Kushi

Table of Contents

Table S1: Distribution of individual chemicals and group sums; median detection limits, quartile cut points, and geometric means with 95% confidence intervals (CIs) (ng/g lipid).

Table S2: Adjusted time ratios (TR) and 95% confidence intervals (CI) of transition to Tanner stage 2+ for individual congener quartiles, compared to Q1.

References

Table S1: Distribution of individual chemicals and group sums[a]; median detection limits[b], quartile cut points, and geometric means with 95% confidence intervals (CIs) (ng/g lipid).

Chemical Congener or Sum (N)[c]	Median MDL	25th %ile	50th %ile	75th %ile	GM (95%CI)	% > MDL
PCB-99	0.5	1.3	2.1	3.5	2.18 (2.06, 2.30)	94.7
PCB-118	1.5	2.1	3.4	5.6	3.48 (3.28, 3.69)	96.7
PCB-153	0.8	3.5	7.2	18.3	7.85 (7.22, 8.54)	98.0
PCB-170	0.5	0.64	1.6	4.8	1.83 (1.67, 2.01)	74.4
PCB-180	0.9	1.5	3.6	11.6	4.19 (3.80, 4.62)	93.3
PCB-138/158	0.6	3.1	5.5	12.5	5.99 (5.54, 6.46)	96.2
Σ_6PCB (n=640)	--	13.3	25.0	57.9	27.3 (25.3, 29.4)	--
PBDE-28	0.6	0.92	1.6	2.5	1.52 (1.43, 1.62)	79.8
PBDE-47	7.8	25.7	42.1	67.5	42.4 (39.8, 45.1)	98.6
PBDE-99	3.6	5.4	9.5	16.1	9.75 (9.13, 10.4)	98.0
PBDE-100	1.9	5.6	9.0	15.4	9.43 (8.86, 10.0)	98.8
PBDE-153	1.9	8.2	13.7	23.9	13.8 (13.0, 14.7)	99.1
PBDE-154	0.6	0.60	1.0	1.6	1.02 (0.96, 1.08)	71.3
Σ_6PBDE (n=639)	--	51.5	78.3	127	82.0 (77.4, 86.8)	--
Hexachlorobenzene (n=641)	2.9	6.4	8.7	11.6	8.67 (8.36, 8.99)	94.7
Trans-nonachlor (n=642)	2.8	2.6	5.9	11.7	5.80 (5.40, 6.23)	68.1
p,p'- DDE (n=642)	39.6	49.9	98.2	190	102 (94.6, 110)	98.6
Σ_3OCP (n=641)	--	63.4	113	216	122 (114, 130)	

Abbreviations: MDL=method detection limit, GM=geometric mean, CI=confidence interval, PCB=polychlorinated biphenyl, PBDE=polybrominated diphenyl ether, DDE=dichloro diphenyl dichloroethene, OCP=organochlorinated pesticide

[a]Other congeners measured and detected at slightly above 60% include: PCB 105, PCB 74, PCB 187, and BDE-85. Additionally measured, but detected at <60%, were PCB101, PCB110, PCB128, PCB146, PCB149, PCB151, PCB156, PCB157, PCB167, PCB172, PCB177, PCB178, PCB183, PCB189, PCB194, PCB195, PCB196_203, PCB199, PCB206, PCB209, PCB28, PCB44, PCB49, PCB52, PCB66, and PCB87; BDE17, BDE183, and BDE66; BB153, p,p’-DDT, o,p’-DDT, β and γ-hexachlorocyclohexane, MIREX, and Oxychlordane (Windham et al. 2010).

[b]Quartile cut-points and GMs are calculated with imputed values for non-detects as used in data analyses, e.g . MDL/√2, whereas median MDL is true value, not converted.

[c]N did not change across PCB or PBDE panels.

Table S2: Adjusted time ratios (TR) and 95% confidence intervals (CI) of transition to Tanner stage 2+ for individual congener quartiles, compared to Q1.

Lipid-Adjusted Congener	Level	Adjusted[a] Breast	Adjusted[a] Pubic Hair	Adjusted[a] with BMI Breast	Adjusted[a] with BMI Pubic Hair
**PCB99**	**Q2**	1.02 (0.99 - 1.05)	1.02 (0.99 - 1.06)	0.99 (0.96 - 1.02)	1.01 (0.98 - 1.04)
	**Q3**	1.04 (1.01 - 1.08) *	1.03 (1.00 - 1.06)	1.01 (0.98 - 1.05)	1.01 (0.97 - 1.04)
	**Q4**	1.04 (1.00 - 1.07) *	1.03* (1.00 - 1.07)	0.98 (0.95 - 1.02)	1.00 (0.97 - 1.04)
**PCB118**	**Q2**	1.02 (0.98 - 1.05)	1.02 (0.99 - 1.05)	0.99 (0.96 - 1.03)	1.01 (0.98 - 1.04)
	**Q3**	1.04 (1.01 - 1.08) *	1.04 (1.00 - 1.07) *	1.01 (0.98 - 1.04)	1.02 (0.98 - 1.05)
	**Q4**	1.04 (1.00 - 1.07) *	1.04 (1.01 - 1.08) *	0.99 (0.96 - 1.03)	1.01 (0.98 - 1.05)
**PCB153**	**Q2**	1.02 (0.99 - 1.06)	1.04 (1.01 - 1.08) **	1.01 (0.98 - 1.04)	1.03 (1.00 - 1.06)
	**Q3**	1.04 (1.00 - 1.07) *	1.06 (1.02 - 1.09) **	1.01 (0.98 - 1.04)	1.02 (0.99 - 1.06)
	**Q4**	1.03 (1.00 - 1.07) *	1.05 (1.01 - 1.08) **	0.99 (0.95 - 1.02)	1.01 (0.97 - 1.05)
**PCB170**	**Q2**	1.02 (0.99 - 1.05)	1.02 (0.99 - 1.06)	1.0 (0.97 - 1.03)	1.01 (0.98 - 1.04)
	**Q3**	1.02 (0.99 - 1.05)	1.04 (1.01 - 1.08) **	0.99 (0.96 - 1.02)	1.02 (0.99 - 1.05)
	**Q4**	1.02 (0.99 - 1.06)	1.04 (1.01 - 1.08) *	0.98 (0.94 - 1.01)	1.00 (0.97 - 1.04)
**PCB180**	**Q2**	1.05 (1.02 - 1.08) **	1.04 (1.01 - 1.07) *	1.02 (0.99 - 1.05)	1.02 (0.99 - 1.06)
	**Q3**	1.05 (1.02 - 1.08) **	1.05 (1.02 - 1.09) **	1.02 (0.99 - 1.05)	1.03 (1.00 - 1.06)
	**Q4**	1.05 (1.01 - 1.08) **	1.06 (1.02 - 1.09) **	0.99 (0.96 - 1.03)	1.02 (0.98 - 1.06)
**PCB138_158**	**Q2**	1.04 (1.01 - 1.07) *	1.04 (1.01 - 1.08) **	1.01 (0.98 - 1.04)	1.02 (0.99 - 1.05)
	**Q3**	1.04 (1.01 - 1.08) *	1.05 (1.01 - 1.08) **	1.01 (0.98 - 1.05)	1.02 (0.99 - 1.06)
	**Q4**	1.05 (1.02 - 1.09) **	1.05 (1.01 - 1.08) **	1.01 (0.98 - 1.04)	1.01 (0.98 - 1.05)
**PBDE28**	**Q2**	1.02 (0.99 - 1.05)	1.02 (0.99 - 1.05)	1.02 (0.99 - 1.05)	1.03 (1.00 - 1.06)
	**Q3**	1.03 (1.00 - 1.06)	1.05 (1.02 - 1.08) **	1.03 (1.00 - 1.06) *	1.05 (1.02 - 1.08) **
	**Q4**	1.02 (0.99 - 1.06)	1.04 (1.01 - 1.07) *	1.02 (0.99 - 1.05)	1.04 (1.01 - 1.07) **
**PBDE47**	**Q2**	1.02 (0.99 - 1.05)	1.0 (0.97 - 1.03)	1.02 (0.99 - 1.05)	1.0 (0.97 - 1.03)
	**Q3**	1.01 (0.98 - 1.04)	1.03 (1.00 - 1.06)	1.02 (0.99 - 1.05)	1.04 (1.01 - 1.07) *
	**Q4**	1.03 (1.00 - 1.07) *	1.04 (1.00 - 1.07) *	1.04 (1.01 - 1.07) *	1.04 (1.01 - 1.07) *
**PBDE99**	**Q2**	1.02 (0.99 - 1.05)	1.0 (0.97 - 1.03)	1.03 (1 - 1.06)	1.0 (0.97 - 1.03)
	**Q3**	1.01 (0.98 - 1.04)	1.03 (1.00 - 1.06)	1.02 (0.99 - 1.05)	1.04 (1.01 - 1.07) *
	**Q4**	1.03 (1.00 - 1.06) *	1.04 (1.01 - 1.07) *	1.04 (1.01 - 1.06) *	1.04 (1.01 - 1.07) *
**PBDE100**	**Q2**	1.05 (1.02 - 1.08) **	1.02 (0.99 - 1.05)	1.06 (1.03 - 1.09) **	1.03 (1.00 - 1.06)
	**Q3**	1.04 (1.01 - 1.07) *	1.03 (0.99 - 1.06)	1.03 (1.00 - 1.06) *	1.03 (0.99 - 1.06)
	**Q4**	1.05 (1.02 - 1.09) **	1.04 (1.01 - 1.08) **	1.06 (1.03 - 1.09) **	1.04 (1.01 - 1.08) **
**PBDE153**	**Q2**	1.05 (1.02 - 1.08) **	1.02 (0.99 - 1.05)	1.04 (1.01 - 1.07) *	1.01 (0.98 - 1.04)
	**Q3**	1.05 (1.02 - 1.09) **	1.03 (0.99 - 1.06)	1.03 (1.00 - 1.06) *	1.01 (0.98 - 1.04)
	**Q4**	1.08 (1.05 - 1.12) **	1.08 (1.04 - 1.11) **	1.04 (1.01 - 1.08) **	1.05 (1.02 - 1.09) **
**PBDE154**	**Q2**	1.00 (0.97 - 1.03)	0.99 (0.96 - 1.02)	1.01 (0.98 - 1.04)	1.00 (0.97 - 1.04)
	**Q3**	1.03 (1.00 - 1.06)	1.03 (1.00 - 1.07) *	1.03 (1.00 - 1.06)	1.03 (1.00 - 1.07) *
	**Q4**	1.00 (0.97 - 1.03)	1.03 (0.99 - 1.06)	1.01 (0.98 - 1.04)	1.03 (1.00 - 1.06) *
**DDE**	**Q2**	1.08 (1.05 - 1.11) **	1.05 (1.01 - 1.08) **	1.06 (1.02 - 1.09) **	1.03 (1.00 - 1.07)
	**Q3**	1.10 (1.06 - 1.14) **	1.07 (1.03 - 1.11) **	1.05 (1.01 - 1.09) *	1.04 (1.00 - 1.08)
	**Q4**	1.10 (1.05 - 1.14) **	1.08 (1.03 - 1.12) **	1.04 (1.00 - 1.08)	1.03 (0.99 - 1.08)
**T_NONA**	**Q2**	0.96 (0.93 - 0.99) *	1.00 (0.97 - 1.03)	0.95 (0.92 - 0.98) **	1.00 (0.97 - 1.03)
	**Q3**	1.00 (0.97 - 1.03)	1.00 (0.97 - 1.04)	0.97 (0.94 - 1.00)	0.99 (0.96 - 1.02)
	**Q4**	1.01 (0.98 - 1.04)	1.04 (1.01 - 1.08) *	0.98 (0.95 - 1.01)	1.02 (0.99 - 1.05)
**HCB**	**Q2**	1.06 (1.03 - 1.10) **	1.04 (1.00 - 1.07) *	1.03 (1.00 - 1.06)	1.01 (0.98 - 1.04)
	**Q3**	1.10 (1.06 - 1.13) **	1.04 (1.01 - 1.07) *	1.05 (1.02 - 1.08) **	1.00 (0.97 - 1.04)
	**Q4**	1.10 (1.06 - 1.13) **	1.06 (1.03 - 1.09) **	1.04 (1.01 - 1.08) *	1.02 (0.98 - 1.06)

Abbreviations: TR=time ratio, CI=confidence interval, PCB= polychlorinated biphenyl, PBDE= polybrominated diphenyl ether, DDE=dichlorodiphenyldichloroethene, HCB=hexachlorobenzene, BMI=body mass index.

[a]Models include Race, Income, Education, Site.

** p<0.01 or * p<0.05 comparied to quartile 1, some interval include 1.0 due to rounding.

References

Windham GC, Pinney SM, Sjodin A, Lum R, Jones RS, Needham LL, et al. 2010. Body burdens of brominated flame retardants and other persistent organohalogenated compounds and their descriptors in U.S. girls. Environmental Research 110, 251–257.
